# Cost-effectiveness of dog rabies vaccination programs in East Africa

**DOI:** 10.1371/journal.pntd.0006490

**Published:** 2018-05-23

**Authors:** Rebekah H. Borse, Charisma Y. Atkins, Manoj Gambhir, Eduardo A. Undurraga, Jesse D. Blanton, Emily B. Kahn, Jessie L. Dyer, Charles E. Rupprecht, Martin I. Meltzer

**Affiliations:** 1 Division of Preparedness and Emerging Infections, National Center of Emerging & Zoonotic Diseases, CDC, Atlanta, Georgia, United States of America; 2 Poxvirus And Rabies Branch, Division of High-Consequence Pathogens and Pathology National Center of Emerging & Zoonotic Diseases, CDC, Atlanta, Georgia, United States of America; Swiss Tropical and Public Health Institute, SWITZERLAND

## Abstract

**Background:**

Dog rabies annually causes 24,000–70,000 deaths globally. We built a spreadsheet tool, RabiesEcon, to aid public health officials to estimate the cost-effectiveness of dog rabies vaccination programs in East Africa.

**Methods:**

RabiesEcon uses a mathematical model of dog-dog and dog-human rabies transmission to estimate dog rabies cases averted, the cost per human rabies death averted and cost per year of life gained (YLG) due to dog vaccination programs (US 2015 dollars). We used an East African human population of 1 million (approximately 2/3 living in urban setting, 1/3 rural). We considered, using data from the literature, three vaccination options; no vaccination, annual vaccination of 50% of dogs and 20% of dogs vaccinated semi-annually. We assessed 2 transmission scenarios: low (1.2 dogs infected per infectious dog) and high (1.7 dogs infected). We also examined the impact of annually vaccinating 70% of all dogs (World Health Organization recommendation for dog rabies elimination).

**Results:**

Without dog vaccination, over 10 years there would a total of be approximately 44,000–65,000 rabid dogs and 2,100–2,900 human deaths. Annually vaccinating 50% of dogs results in 10-year reductions of 97% and 75% in rabid dogs (low and high transmissions scenarios, respectively), approximately 2,000–1,600 human deaths averted, and an undiscounted cost-effectiveness of $451-$385 per life saved. Semi-annual vaccination of 20% of dogs results in in 10-year reductions of 94% and 78% in rabid dogs, and approximately 2,000–1,900 human deaths averted, and cost $404-$305 per life saved. In the low transmission scenario, vaccinating either 50% or 70% of dogs eliminated dog rabies. Results were most sensitive to dog birth rate and the initial rate of dog-to-dog transmission (Ro).

**Conclusions:**

Dog rabies vaccination programs can control, and potentially eliminate, dog rabies. The frequency and coverage of vaccination programs, along with the level of dog rabies transmission, can affect the cost-effectiveness of such programs. RabiesEcon can aid both the planning and assessment of dog rabies vaccination programs.

## Author summary

Dog rabies causes, globally, approximately 55,000 human deaths per year. Mass vaccination programs can control dog rabies. We built a spreadsheet-based tool, RabiesEcon, to aid public health officials in planning large-scale dog rabies vaccination programs. We used RabiesEcon to estimate the cost-effectiveness of dog rabies control programs in East Africa for a human population of one million (approximately 2/3 urban, 1/3 rural). We evaluated three different vaccination options: no vaccination, annual vaccination of 50% of dogs, and semi-annual vaccination of 20% of dogs. Over a 10-year period, no dog vaccination results in approximately 44,000–65,000 rabid dogs and 2,000 human deaths. Annually vaccinating 50% of dogs for 10 years resulted in approximately 42,000–48,000 fewer rabid dogs and approximately 2,000–1,600 fewer human deaths. These reductions cost approximately $450-$385 per life saved. Semi-annual vaccination of 20% of dogs for 10 years resulted in approximately 41,000–50,000 fewer rabid dogs and approximately 2,000–1,900 fewer human deaths. These reductions cost approximately $400–$300 per life. In certain scenarios, 70% of dogs vaccinated eliminated dog rabies. Dog rabies vaccination programs can control, and potentially eliminate, dog rabies.

## Introduction

Rabies causes an estimated 25,000–70,000 human deaths annually, with about 90% of those deaths due to dog rabies [1–2]. Human rabies can be prevented through prompt post exposure prophylaxis (PEP) [3–5]; however, human rabies vaccine and immune globulin, needed for PEP, are frequently unavailable or unaffordable in developing countries with the highest burden of human rabies exposure [3, 6]. Controlling dog rabies through large-scale dog vaccination programs effectively reduces human rabies mortality [7–10].

Previous studies have modeled dog rabies transmission and probabilities of human death after contact with a rabid animal [7, 8, 11–16], as well as estimating the cost effectiveness of specific dog rabies control programs [7, 17–21]. There are few tools available, however, that public health decision makers can readily use to estimate the impact and the cost-effectiveness of dog rabies control programs in their jurisdictions. We extend the existing literature by presenting an easy-to-use spreadsheet-based tool, called RabiesEcon, which public health officials can use to calculate the costs-and-benefits of dog rabies vaccination programs, including the number of averted rabid dogs and human rabies cases. We use RabiesEcon to estimate the impact and cost-effectiveness of dog rabies vaccination programs in a representative East African population of 1 million. Input values can be readily changed to represent almost any country or region, and thus RabiesEcon can provide public health officials with essential data for decision making related to controlling dog rabies.

## Methods

### Overview

RabiesEcon is a spreadsheet-based tool (S1 Appendix) that incorporates a mathematical (deterministic) model of dog-dog and dog-human rabies transmission to estimate dog and human rabies cases averted, and the cost per human rabies death averted and per year of life gained (YLG) due to dog rabies vaccination programs. We used RabiesEcon to estimate the cost-effectiveness of dog rabies vaccination programs in an illustrative East Africa human population of 1 million in a mixture of urban and rural settings. Because there are insufficient data from a single country in Africa for every input in RabiesEcon, we used data from a number of African countries, primarily Chad, Malawi and Tanzania (Table 1). We estimated, based on published measurements of dog ownership in East Africa [2, 20], that the modeled population has approximately 82,000 dogs (36,500 in urban setting, 45,700 in rural setting) (Table 1). We chose East Africa as an example because recently published studies demonstrated the feasibility of conducting dog rabies vaccination programs in this region [20–23]. We built RabiesEcon to include a separate sub-model for each sub-region, urban and rural. Each sub-model calculates the number of dog rabies, human deaths and impact of dog vaccinations and PEP for that sub-region, using data relevant to the sub-regions (Table 1). The results from each sub-region are then summed and presented as a total for the entire area being studied.

**Table 1:** Main demographic and epidemiological model inputs to estimate the cost-effectiveness of an illustrative dog rabies vaccination programs in East Africa.

Model Variable	Model Values		Source
	Urban	Rural	
**Human population**			
Total Human Population	661,444	338,047	[25]
Square kilometers (km^2^)	220	1,792	[25]
Human Population (per km^2^)	3,007	189	Calculated^a^
Human birth rate (per 1,000 pop)	36.0	39.8	[26]
Human life expectancy(yrs)	58.5	58.5	[27]
Life expectancy at age 10 yrs	52.8	52.8	[27]
Average age of death due to dog rabies (yrs)	10	10	[2]
Estimated annual human deaths from dog rabies at the beginning of the program	20	10	[1,2,20]
**Dog population**			
Number of humans-per-dog^b^	18.1	7.4	[2, 20, 30]
Total Dog Population	36,544	45,682	Calculated^a^
Dog per km^2^	166	25.5	Calculated^a^
Dog birth rate (per 1,000 dogs)^c^	676	572	[7,29]
Dog life expectancy, years^d^	3	3	[29]
Probability of clinical outcome (dogs)	0.45	0.45	[8]

^a^ Calculated using the RabiesEcon tool. Please see Supplemental material.

^b^ The numbers of humans-per-dog for Blantyre were obtained from Gibson et al. [20]; the estimate for rural areas was based on Knobel et al.’s estimate for Africa [2].

^c^ The urban dog birth rate was obtained from a dog population household survey in N’Djamena, Chad [7]. For the rural scenario, we used data from Machakos District, Kenya [28].

^d^ Life expectancy at birth was 3.5 and 2.4 years for male and female dogs in Kenya [29].

We compared three different dog rabies vaccination options: no vaccination, annual vaccination of 50% of all dogs, and semi-annual vaccination of 20% of dogs. We included, for each vaccination option, two dog rabies transmission scenarios: low (1.2 dogs infected per infectious dog) and high (1.7 dogs infected per infectious dog) (see later for further details). We used several published sources of demographic, epidemiological, and economic data (Table 1). We used a government perspective (government-as-payer). We assessed the impact of the interventions over a 10-year period, and we discounted all future costs and benefits (including lives saved) at a rates of 3% and 16% [24]. The later discount rate was derived from the weighted average yield to maturity for 10-year Bank of Tanzania Treasury bonds in October 2017. (https://www.bot.go.tz/financialmarkets/aspSmartUpload/TBondsResults.asp: accessed May 10, 2018). A user of RabiesEcon can alter almost all the input values.

### Demographic and epidemiological inputs

Our illustrative East African example includes urban and rural settings, using a population of approximately 1 million, with 2/3 of that population in an urban setting and 1/3 in a rural setting (Table 1). We set the total area occupied by this population at approximately 2,000 sq. km., with approximately 200 sq. km. being urban (Table 1). These urban and rural settings allow for differences in human and dog population densities, and resultant differences in risk of rabies transmission (Table 2). We used, based on published studies, a rate of human to dog population of 18.1:1 for the urban areas and 7.4:1 for the rural areas [2, 20, 30] (Table 1).

**Table 2:** Values to estimate dog-to-dog rabies transmission in East Africa.

	A. Low dog-to-dog rabies transmission		B. High dog-to-dog rabies transmission	
Dog-dog rabies transmission parameter	Urban	Rural	Urban	Rural
Bites per rabid dog to another dog	2.7	2.4	3.8	3.1
Calculated average rabies cases generated from an infectious rabid dog, at steady state ^a^	1.2	1.1	1.7	1.4

^a^ The number of dogs infected per infectious dog is sometimes termed as the basic reproduction number, R_0_. The biting behavior of rabid dogs during the course of infectious periods in rural Tanzania was highly variable (mean bites per rabid dog = 2.15, standard deviation: 2.37) [8].

### Transmission model and assumptions

We used a previously published model [7] as a basis for our mathematical model of rabies transmission incorporated into RabiesEcon (for equations, see S2 Appendix). We provide in Tables 1 and 2, and S2 Appendix (Table 1), a list of inputs used in the transmission model. The model uses one-week time steps. The introduction of rabies into a previously uninfected dog population initially results in large oscillations in the estimated weekly number of rabid dogs. We therefore, to make it easier to facilitate comparisons between no vaccination and dog vaccination programs, programmed into RabiesEcon a process to calculate a “steady state” of a near-constant number of annual cases of canine rabies in a “no vaccination” scenario. We did this by programming RabiesEcon to run an initial 10,000 weeks (S2 Appendix and Table 1 shows the specific parameters used).

Because the risk of dog rabies transmission depends on a number of variables, such as the density of dogs and bites per rabid dog when attacking susceptible dog, we included in our analyses of each vaccination program two scenarios, low and high, of rates dog-to-dog rabies transmission [8,12,30]. We calculated the number of dogs infected per infectious dog as follows:

Number of dogs infected per infectious dog (Ro) = Number of bites from infectious dog to susceptible dog x risk of infection per bite from infectious dog.

Based on data from Tanzania, we used a range of 2.4–3.8 bites per infectious dog [8]. We then, to provide a range of Ro values from 1.1 to 1.7 (Table 2), assumed a value of 0.45 as the risk of infection per bite from infectious dog (S2 Appendix. Table 1). The range of values of Ro used closely follows the range reported by Hampson et al [8], when they reviewed the literature of canine rabies transmission dynamics. The number of dogs infected by an infectious dog (Ro value) is likely impacted by factors such as dog density and percentage of dogs that are unconfined (free roaming). The relationship between those and Ro is not well measured. Thus, any value chosen or calculated becomes a proxy for the impact of those other factors.

We note that deterministic models, of the type used to build RabiesEcon, allow for the number of infectious dogs to be reduced to less than 1 (e.g., 0.5 infectious dog), but still able to transmit. This can result in “pop up” outbreaks of dog rabies in later years. We retained this factor for two reasons; It can be interpreted as mimicking, to a degree, the risk of importation of a rabid animal from outside, or the incomplete recording of all rabid dogs within, the dog rabies control area. And, users of RabiesEcon can easily ignore those “pop-up” outbreaks that occur in years well beyond the chosen analytic horizon (e.g., if the user runs a scenario in which dog rabies is eliminated by year 6, “pop up” of cases in, say, year 16 can be assumed to be due to the mechanics of the model).

### Interventions

As stated earlier, we compared a no vaccination option to two dog vaccination options (annual vaccination of 50% of all dogs, and semi-annual vaccination of 20% of dogs) (Table 3). The 50% annual coverage rate reflects, approximately, the average rate found by Jibat et al when they reviewed dog rabies vaccination coverage in Africa as reported in 16 published papers [31]. The 20% rate for semi-annual vaccination represents a potentially cheaper alternative (i.e., 10% less dogs are vaccinated). However, because the high turnover of dog populations (due to a combination of short life expectancy and high dog birth rate–Table 1), an annual vaccination program may result in up to 1/3 of vaccinated dogs dying in the interim between vaccinations programs. A smaller, but more frequent, semi-annual vaccination program may result in almost the same percentage of vaccinated dogs as with the annual program.

**Table 3:** Characteristics of the mass dog vaccination and neutering programs, and post-exposure prophylaxis.

Item	No mass vaccination	Mass vaccination Programs		Source
		Option 1	Option 2	
Frequency of vaccination^a^	None	Annual	Biannual	Assumed
Vaccination program coverage^b^	0%	50%	20%	Assumed
Dog vaccine effectiveness^c^	N/A	95%	95%	[4]
Weekly loss vaccine immunity (wks 0–25)	N/A	0.81%	0.81%	[7]
Weekly loss vaccine immunity (wks 26–52)	N/A	11.1%	11.1%	[7]
Female dogs spayed, annual^d^	0%	0%	0%	[29]
Male dogs neutered, annual^d^	0%	7.5%	7.5%	[29]
Laboratory testing of dogs	0.7%	0.7%	0.7%	[19]
Bite investigation	5%	5%	5%	[19]

a. Frequency of vaccination: number of times the vaccination is given in a year.

b. Vaccination coverage: percent vaccinated each time the vaccine is given. Option 1 considers annually vaccination covering 50% of the dog population. In Option 2 considers biannual vaccination covering 20% of the dog population during each vaccination program.

c. Assumed that rabies vaccine in dogs is the same level of effectiveness as in humans [4].

d. We assumed that the percentage of dogs neutered would be half that observed in 150 dog owning households Machakos, Kenya [29]. See text for further details.

These dog vaccination Options are illustrative, and can be readily changed by a user. We examine, in the sensitivity analysis, the impact of increasing the vaccination rate to the World Health Organization recommended level of 70% [2, 3,12]. We assumed that dog rabies vaccine, when correctly administered, was 95% effective, similar to the effectiveness in humans [4]. Following Zinsstag et al. [7], we included waning immunity in dogs vaccinated against rabies (Table 3).

Because dog birth rate greatly influences dog-to-dog rabies transmission [7, 29], we included in the dog vaccination options concurrent dog population control programs, in which annually 7.5% of the intact male dogs were neutered (Table 3). We assumed that, for a user-defined percentage of male dogs neutered, there will be an equal percentage reduction in the number of dog litters, and thus a reduced dog population. We based this percentage on half the percentage of castrated male dogs observed in a survey of 150 dog-owning households in Machakos, Kenya [29]. We halved the percentage observed in Machakos because that was a relatively small survey, and our experience is that dog neutering programs in Africa are frequently under-resourced and thus do not impact large portions of the dog populations. We altered this assumption in our sensitivity analysis (see later).

We assumed, based on recent data from Haiti (which faces rabies control resource constraints similar to many countries in Africa), that dogs with rabies symptoms would be immediately euthanized, and a small percentage (0.7%) of the brains from those animals would be laboratory tested for rabies (Table 3). We further assumed that 5% of all dog-human bites would be investigated for potential rabies transmission [19]. Finally, we assumed that 21% of dog bite victims would start post-exposure prophylaxis (PEP) (see later, Table 4). We assumed a 95% efficacy when PEP is given as per recommended protocols, [4]. We altered in our sensitivity analyses the percentage of dog bite victims who receive PEP (see later).

**Table 4:** Human and animal costs related to treating suspected rabies exposures and dog population management^a^.

Materials	Value	Source
**Human post exposure prophylaxis (PEP)**		
Probability receiving PEP^b^	21%	[19]
Human vaccine efficacy (%)^c^	0.95	[4]
Material cost ($/dose)	0.40	[21]
Overhead cost per visit ($/visit)	2.10	[2,24]
Cost per vaccine (tissue-culture) ($/dose)	12.33	[2]
Number of vaccines (per visit)	1	[3]
Number of visits required for PEP regime	5	[3]
**Rabies immunoglobulin (RIG)**		
Proportion of PEP patients receiving RIG	7%	[2,19]
Average cost of RIG ($)	135.59	[2]
**Average cost of patient PEP**^**d**^		
Average cost of PEP ($)	83.65	Calculated
**Dog laboratory testing, bite investigation, and population management**		
Laboratory testing ($/dog)	6.79	[2]
Bite investigation ($/dog)	20.61	[19,35]
Weighted average cost of laboratory tests and bite investigation ($/dog suspected rabies)^e^	1.08	Calculated
Spayed dog ($/dog)	8.00	[18,36]
Neutered dog ($/dog)^f^	3.40	[37,38]

a. We used a 3% discount rate [24]. All costs adjusted to 2015 US dollars [32].

b. Percent of exposed humans who receive PEP and are fully compliant with PEP treatment regime such that they are protected against developing rabies. The percentage receiving PEP is regardless of dog vaccination option considered. The 21% estimate comes from a recent study in Haiti [19], where out of the 54% of bite victims who sought medical care, only 39% began PEP.

c. Vaccine efficacy estimated at approximately 95%, if guidelines for dose schedule are followed [4].

d. Costs per patient receiving PEP (Table 3). Cost of PEP includes costs of materials (needles, swabs, etc.), tissue-culture vaccine, RIG (7% of patients receiving PEP receive RIG), and costs of 5 visits to a public health facility.

e. Weighted average cost calculated as follows: (probability of bite investigation x $ of bite investigation) + (probability of laboratory testing x $ of laboratory testing). Probabilities from Table 3.

f. The material costs of $2.22 per castrated dog [38]. We added, for each castrated dog, $0.65 for human resources, $0.24 for awareness programs, and $0.29 for transportation [37]. See Table 3 for description of coverage of neutering programs

We used, when modeling the dog vaccination strategies, the following three assumptions. Dog rabies is endemic (i.e., near steady state) in the region being analyzed. Second, mass vaccination campaigns last 10 weeks, each year (or 10 weeks twice per year if bi-annual). Third, the dog population can only increase to a maximum of 5% per year, which is near the lower limit measured by Kitala et al. in Machakos District, Kenya [28]. Kitala et al stated that the dog population in Machakos was growing at a rate faster than normally encountered in Africa.

### Outcomes and cost-effectiveness

We calculated the cumulative 10-year totals of the number of rabid dogs, human rabies deaths and YLG with and without the rabies vaccination programs. We also estimated the 10-year total cost of each program. To calculate the cost-effectiveness over 10 years of each vaccination option per human death averted, we used the following formula:

$$\mathrm{Cost}\;\mathrm{per}\;\mathrm{human}\;\mathrm{death}\;\mathrm{averted}=\frac{\mathrm{Costs}\;\mathrm{of}\;\mathrm{dog}\;\mathrm{vaccination}\;\mathrm{program}-\mathrm{costs}\;\mathrm{incurred}\;\mathrm{with}\;\mathrm{no}\;\mathrm{vaccination}\;\mathrm{program}}{\mathrm{Number}\;\mathrm{of}\;\mathrm{human}\;\mathrm{deaths}\;\mathrm{without}\;\mathrm{vacciantion}\;\mathrm{program}-\mathrm{human}\;\mathrm{deaths}\;\mathrm{with}\;\mathrm{vaccination}\;\mathrm{program}}$$

For estimates of cost per case averted over more than 1 year (e.g., 10 years), each component of the formula was first summed, then the overall result calculated (e.g., for a 10 year cost of human death averted, the 10 year cost for dog vaccination program was summed separately, then added into the formula). When discounting was applied, each component was individually discounted to year 1.

We used a similar formula to calculate the cost per YLG, assuming that the average age of dog-rabies related death is 10 years of age [28], and that life expectancy at age 10 is approximately 53 years [27] (Table 1) (Additional details in S2 Appendix, Note #2).

### Cost inputs

#### Overview

We included, when estimating the costs of dog vaccination programs, the costs of treating humans suspected of rabies exposure, cost of dog population management, and the costs of mass dog vaccination. As previously stated, we used a government perspective (government-as-payer), and thus we did not include costs borne by the patient, such as co-paid medical bills or time lost from work. In addition to the previously mentioned discounting, we adjusted all costs to 2015 US dollars using US gross domestic product implicit price deflators [32].

#### Costs associated with suspected rabies exposures

We, assumed, based on data from Haiti, that just 21% of exposed persons receive PEP [19]. There are very few studies reporting the probability that a dog bite victim receives PEP [1]. Hampson et al. estimated the probability of receiving PEP as function of the Human Development Index (HDI) [1]. An exposed person in a country with an HDI of 0.3–0.5 (on a scale of 0 to 1, with 1, with 1 being the ideal) had an approximate probability of receiving PEP of 0.4 to 0.8. However, data from Haiti indicate that only 1/3 of those who receive PEP are fully compliant [19]. We also conducted sensitivity analyses in which we examined the impact of assuming the 99% of all potential dog rabies exposures receive PEP (see later).

We estimated an average cost of $83.65 per person receiving PEP due to suspected rabies exposure (Table 4). This cost includes materials (needles, swabs, etc.), tissue-culture vaccine, and cost per outpatient visit to a public health facility (Table 4). The use of rabies immunoglobulin (RIG) in most countries with high burdens of rabies is negligible due to high relative costs and limited supply [1,33, 34]. We assumed that 7% of patients receiving PEP would receive RIG. This assumption was based on Knobel et al.’s estimate of 1% of PEP patients received RIG usage [2], and data from Haiti that 13% of patients receiving PEP also received RIG [19].

#### Costs of dog management and laboratory testing

Recommendation for quarantining and testing dogs that have bitten a person vary depending on local rabies prevalence and national recommendations [4, 39]. We estimated, using the probabilities of laboratory testing of dogs suspected of having rabies and bite investigations (Table 3), an average cost of $1.08 per dog for laboratory testing and bite investigations (Table 4).

#### Cost of dog neutering and spaying

We calculated a cost of $3.40 per neutered male dog (Table 4). We based this cost on the cost of $2.22 for pinhole castration in Uganda [38]. To the Uganda-based cost data, we added $0.65/dog for human resources, $0.24/dog per awareness program, and $0.29/dog per transportation costs. We based these non-medical costs using data from a dog vaccination program in Chad [37]. For comparison, the costs associated with a standard surgical castration of puppies in Uganda were $6.02 [40]. Note, that although we did not incorporate in this example the spaying of female dogs, such an option can be selected in RabiesEcon. The cost of spaying, however, is typically greater than neutering (Table 4).

#### Vaccination program costs

We used an average cost per dog vaccinated of $2.39 (Table 5). We based this on previous studies of mass dog vaccination programs in East Africa [21,23,37,40] (Table 5, and S2 Appendix, Table 2). Operating costs included training, public awareness and program information (e.g., media such as posters and advertisement), personnel costs (e.g., costs of supervisors, technicians, general staff), transportation (i.e. vehicles, gasoline), and other equipment. Medical supply costs included supplies such as dog rabies vaccines, syringes, needles, animal marking, and vaccination certificates. For comparison, Elser et al reviewed published costs of dog rabies vaccination, and found a range $1.13/ dog vaccinated in Bhutan to $5.41/ dog in Kwa-Zululand, South Africa, with upper limits at approximately $11–$16/ dog for different phases of vaccination programs in southeastern Tanzania [41].

**Table 5:** Mass dog rabies vaccination program costs and average costs per dog vaccinated^a^^,^^b^.

Item	No mass vaccination	Mass vaccination^c^		Source
		Option 1 Annual vaccination program	Option 2 Twice yearly vaccination program	
Vaccine administration	N/A^d^	35,504	28,403	[21,36,37,40]
Workers at vaccination site	N/A	26,616	21,293	[21,36,37,40]
Transportation	N/A	17,496	13,997	[21,36,37,40]
Miscellaneous materials	N/A	18,644	14,915	[21,36,37,40]
Total cost	N/A	98,260	78,608	Calculated
Average cost per dog vaccinated	N/A	2.39	2.39	Calculated

a. See Table 3 for description of frequency and coverage of vaccination programs.

b. Additional details in S2 Appendix, Table 2.

c. Mass vaccination options are either once-per-year (Option 1) or twice per year (Option 2). See Table 3 and main text further description.

d. N/A = not applicable.

### Sensitivity analyses

In addition to presenting all our results based on two different scenarios of low and high dog-to-dog rabies transmission (Table 2), we conducted the following sensitivity analyses. First, we examined the impact on estimates of rabid dogs in the high transmission scenario by changing the percentage of dogs neutered during the vaccination programs from 7.5% (Table 3) to either 0% or 20%, assuming use of vaccination Option 1 (50% dogs vaccinated annually). Second, we calculated the number of rabid dogs if 0%, 20%, 50%, and 70% of the dog population were vaccinated annually, over a 30-year period. The 70% level is the World Health Organization (WHO) recommended minimum level of rabies vaccination needed to ensure dog rabies elimination [2, 3,12].

We also considered the value of increasing PEP coverage from the base case of 21% (Table 3) to 99%. Assuming that the effectiveness of PEP is 95% (Table 4), and that all those exposed comply with the full PEP regime, such a strategy would be designed to prevent almost all loss of human life to dog rabies, without the cost of large-scale dog rabies vaccination programs. Because such a strategy would have to continue without cessation due to the unceasing threat of rabid dogs, we calculated the results for both 10 years (as for the other analyses in this paper), and for 30 years.

Finally, we noted that the rate of onward dog-to-dog transmission is a crucial factor in estimating the spread of dog rabies and the consequent benefits of vaccinating dogs against rabies. We therefore conducted a multivariable analysis in which we made simulations changes in the following 4 variables that most directly impact the number of rabid dogs in our scenarios (Table 1). Annual percentage dogs vaccinated (30%, 40%, 50%—baseline 50%); Dog birth rate (550 and 350/1,000 dogs–baseline 676/1,000); Dog life expectancy (3.0 and 2.5 years–baseline 3.0 years); and, initial rate of dog-to-dog transmission, Ro (1.2, 1.5, 1.8 –baseline 1.2). To simplify, when running this sensitivity analysis, we only used the values for the “urban” setting (Table 1) (i.e., “turned off” rural settings).

The range of annual percentage of dogs vaccinated was based on observations that these are the levels of coverage need to begin to observe “notable” reductions, but not guaranteed elimination, of human rabies deaths [1]. The estimate birth rate of 550/1,000 dogs was based on the lower 99% confidence interval from N’Djamena, Chad [7]. The lower estimate of 350/1,000 dogs came from birth rates for young dogs (≤ 12 months of age) in rural Machakos District, Kenya [29]. The lower estimate of life expectancy is based on data from N’Djamena, Chad [7]. The Ro values examined are similar to those in Table 2, which we derived from the review by Hampson et al. [8].

## Results

### Health outcomes

**Fig 1:**
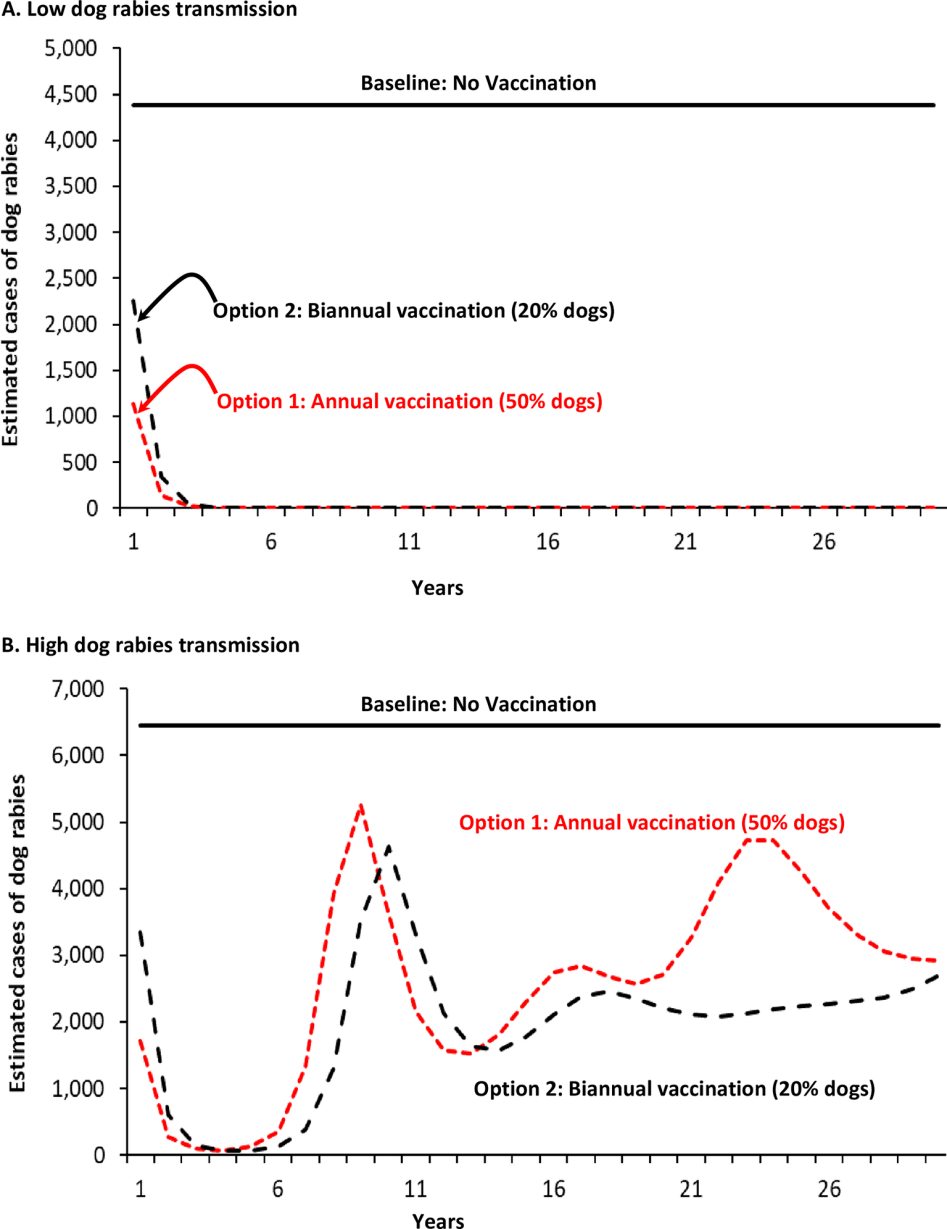
Cases of dog rabies for alternative dog rabies vaccination programs in East Africa: A. Low levels of transmission; B. High levels of transmission^a^ Footnotes: a. Results for two scenarios for dog rabies vaccination programs in an East African population of 1 million persons (approximately 2/3 urban, 1/3 rural), with approximately 82,000 dogs (Table 1). Vaccination programs: Option 1, annual mass dog vaccination, resulting in 50% of the dog population vaccinated, and Option 2, biannual (twice per year) mass dog vaccination, resulting in 20% of the dog population vaccinated for each vaccination program. Rabies transmission risk is defined, in part, by the number of bites per rabid dog to another dog (see Table 2).

Without a vaccination program, in the illustrative example there would be approximately 4,500 (low rabies transmission) to 6,500 (high transmission) rabid dogs per year, totaling approximately 44,000–65,000 rabid dogs over ten years (Fig 1 and Table 6). In the low rabies transmissions scenario, dog rabies vaccination options resulted in almost complete control of dog rabies within 5 years, with 10 years total reductions of approximately 42,600–41,200 rabid dogs, for dog vaccination Options 1 and 2 respectively (Fig 1 and Table 6). Such control remained for more than 10 years (assuming the vaccination programs continued) (Fig 1). In the high transmission scenario, the 10 year total reductions of rabid dogs were approximately 47,800–50,300, for vaccination programs Options 1 and Options 2, respectively (Table 6). Dog rabies cases begin to increase, for both options, at year 6, and thereafter the number of cases fluctuates, albeit always lower than “no vaccination” option (Fig 1). Note that, in the high transmission scenario, vaccination Option 2 results in fewer rabid dogs, despite a lower total of dogs vaccinated (Fig 1, Table 6). This is because, with the relatively high birth rate and short life spans of dogs in East Africa (Table 1), more frequent vaccination programs (i.e., twice per year) protect a relatively larger portion of living dogs (i.e., dogs are vaccinated closer to the time of their birth, and thus here is a smaller pool of dogs susceptible to rabies).

**Table 6:** Ten-year cumulative health outcomes, program costs and cost-effectiveness for alternative dog rabies vaccination programs in East Africa, by risk of rabies transmission.^a^.

Item	No vaccination	10 year impact of vaccination programs: Per million population ^a^^,^ ^b^	
		Option 1: 50% dog population, annual	Option 2: 20% dog population, biannual
	Rabies dog-dog rabies transmission risk ^c^		
	Low-high	Low-high	Low-high
**A. Effectiveness of the intervention (undiscounted)**			
Number of rabid dogs	43,868–64,533	1,307–16,771	2,669–14,192
Number of rabid dogs averted	NA	42,562–47,762	41,200–50,341
Percentage reduction in rabid dogs	NA	97%-74%	94%-78%
Human rabies deaths	2,132–2,904	63–1,263	126–964
Human rabies deaths averted	NA	2,069–1,641	2,006–1,940
Percentage reduction in human deaths		97%-57%	94%-67%
Years of life gained (YLG)	NA	118,269–97,364	112,382–110,150
**B. Cost of the intervention (undiscounted, US$)**			
Dog vaccine administration (include biologics)	0–0	1,128,954–960,160	897,406–767,903
Spade and Neuter costs	0–0	120,453–102,444	119,685–102,414
Post-exposure prophylaxis (PEP)	410,751–563,894	112,107–157,562	216,759–300,418
Investigation suspected rabid dog costs^d^	25,207–34,605	6,880–9,669	13,302–18,436
Total costs	435,958–598,499	1,368,394–1,229,835	1,247,152–1,189,171
**C. Average cost-effectiveness compared to no mass vaccination**			
** Undiscounted**			
Cost per human death averted (US$/death)	NA	451–385	404–305
Cost per year of life gained (US$/YLG)	NA	8–6	7–5
**Discounted at 3%**^**e**^			
Cost per human death averted (US$/death)	NA	460–368	415–299
Cost per year of life gained (US$/YLG)	NA	17–13	16–11
**Discounted at 16%**^**e**^			
Cost per human death averted (US$/death)	NA	509–327	468–290
Cost per year of life gained (US$/YLG)	NA	103–64	98–60

a. Results for two scenarios for dog rabies vaccination programs in an East African population of 1 million persons (approximately 2/3 urban, 1.3 rural), with approximately 82,000 dogs (Table 1).

b. Vaccination programs: Option 1, annual mass dog vaccination, resulting in 50% of the dog population vaccinated, and Option 2, biannual (twice per year) mass dog vaccination, resulting in 20% of the dog population vaccinated each time a vaccination program is run.

c. Rabies transmission risk is defined by number of bites per rabid dog to another dog (see Table 2).

d. Suspect rabies exposure costs are those costs associated with investigating a dog suspected of having rabies and investigating any bites on humans associated with that animal. Further details, see Tables 3 and 4 and main text.

e. 3% and 16% discount rate applied to both health outcomes and costs. US$ denotes 2015 US dollars. The 16% discount rate was derived from the weighted average yield to maturity for 10 year Bank of Tanzania Treasury bonds in October 2017. (https://www.bot.go.tz/financialmarkets/aspSmartUpload/TBondsResults.asp: accessed May 10, 2018)

**Fig 2:**
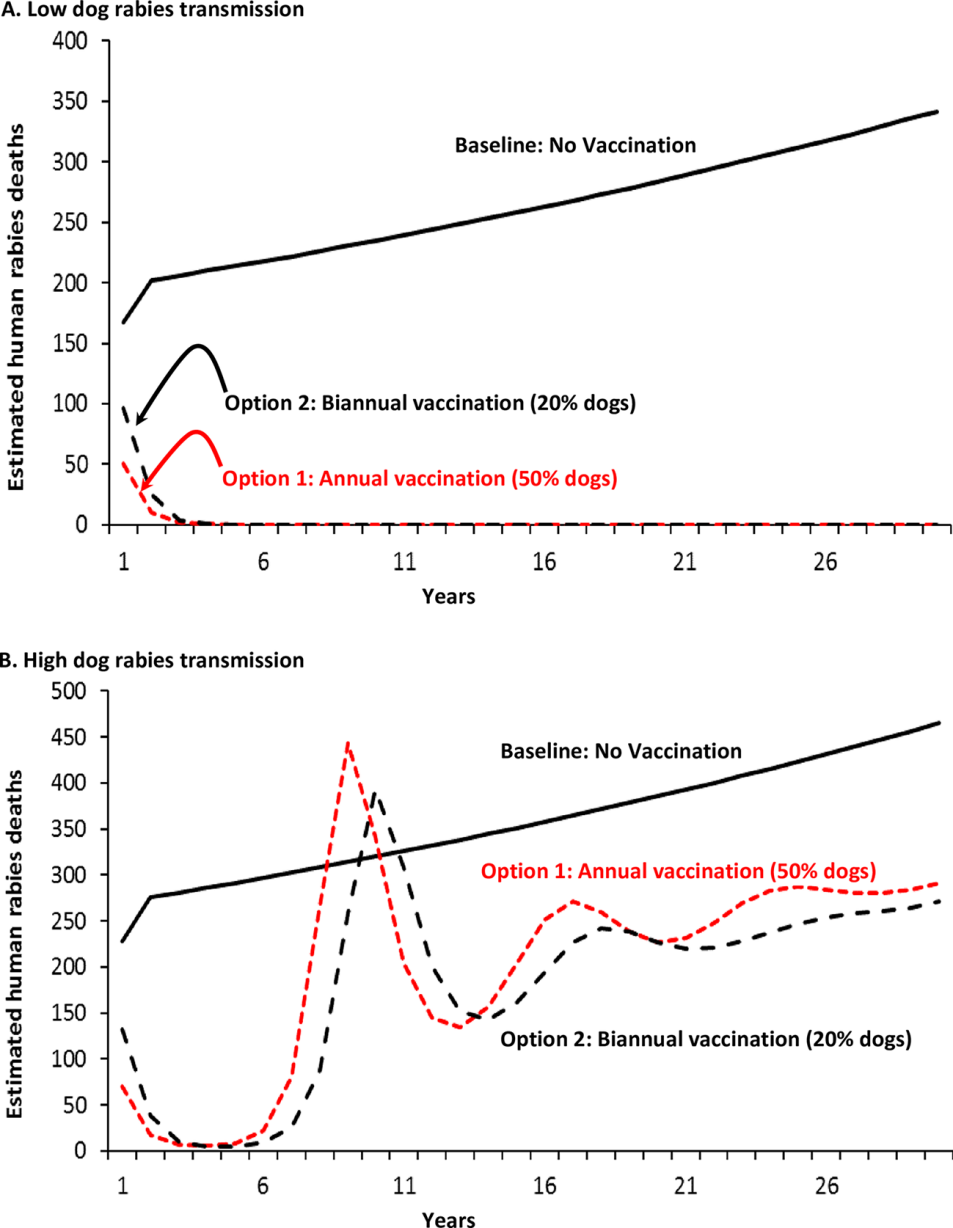
Cases of human rabies per million human population for alternative dog rabies vaccination programs in East Africa: A. Low levels of transmission; B. High levels of transmission^a^ Footnotes: a. Results for two scenarios for dog rabies vaccination programs in an East African population of 1 million persons (approximately 2/3 urban, 1/3 rural), with approximately 82,000 dogs (Table 1). Vaccination programs: Option 1, annual mass dog vaccination, resulting in 50% of the dog population vaccinated, and Option 2, biannual (twice per year) mass dog vaccination, resulting in 20% of the dog population vaccinated for each vaccination program. Rabies transmission risk is defined, in part, by the number of bites per rabid dog to another dog (see Table 2).

Human rabies deaths, without a vaccination program, total approximately 2,100–2,900 over 10 years (Table 6). The impact of vaccination programs on human deaths follows the same pattern as that for numbers of rabid dogs (Fig 2). The number of human deaths averted, under low rabies transmissions scenario, range from 2,100–2,000 for vaccination programs Options 1 and 2, respectively. The deaths averted under the high transmission scenario range from approximately 1,600 (Option 1) and 1,900 (Option 2) deaths averted (Table 6). The reason why more deaths are averted in the high transmission scenario with vaccination option 2, compared to vaccination Option 1, is the same as the previously given explanation for the relatively lower number of dog rabies cases occurring under the same vaccination program (Table 6).

The 10-year total program cost for dog vaccination Option 1 (annual vaccination of 50% of the dog population) was $1.4 million to $1.2 million, and Option 2 (20% of the dog population vaccinated) cost $1.2 million to $1.2 million (Table 6). The no vaccination option would cost the government, over 10 years, approximately $0.4 million to $0.6 million. The undiscounted 10 year cost-effectiveness for Option 1 vaccination program ranged from $451-$385 per death averted (low and high rabies transmission, respectively) and $8-$6 per YLG (Table 6). The undiscounted cost-effectiveness for vaccination Option 2 were similar (Table 6).

### Sensitivity analysis

**Fig 3:**
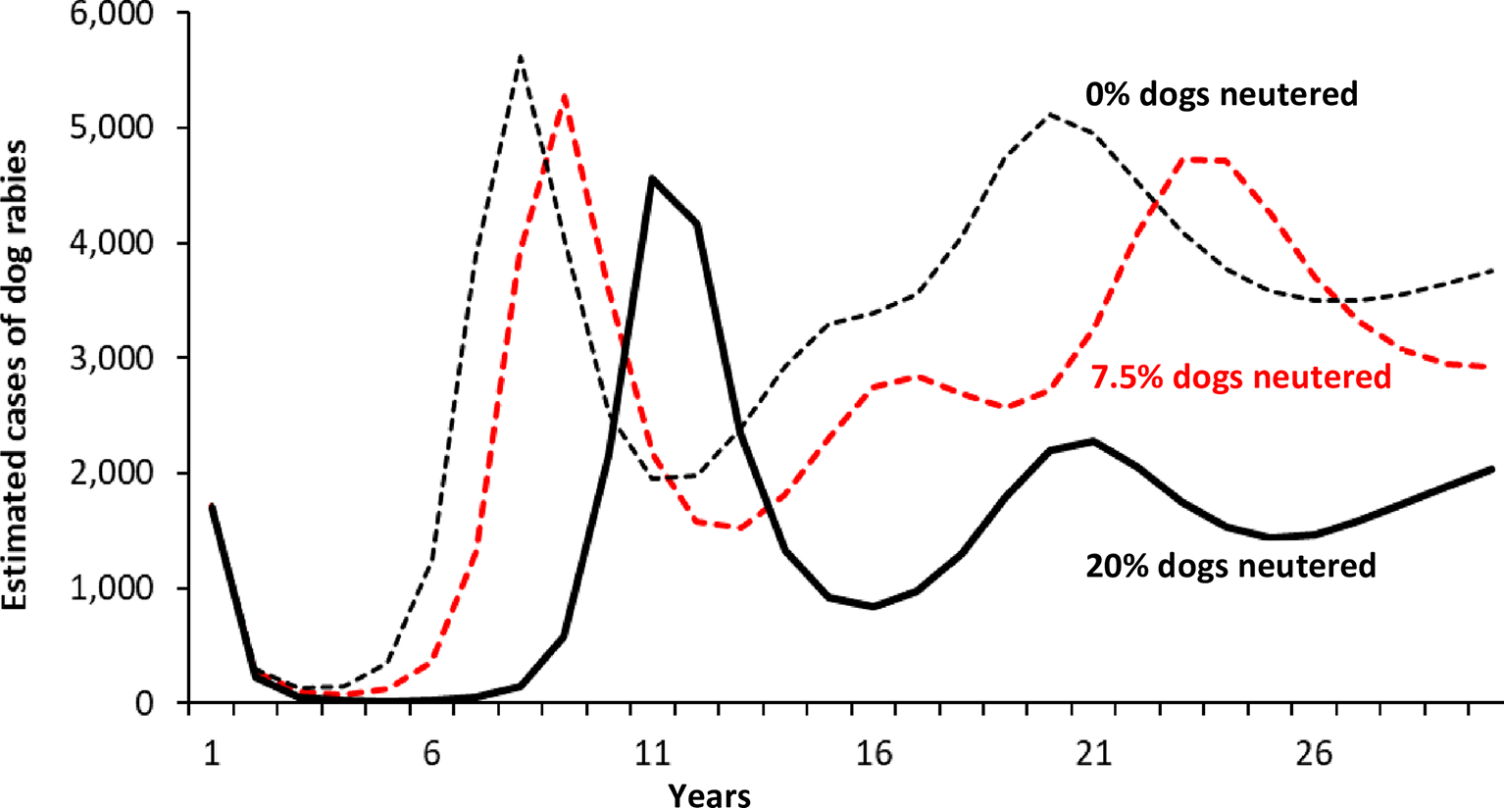
Sensitivity analysis: Estimated number of dog rabies cases for different proportions of male dogs neutered^a^ Footnotes: a. Results estimated assuming an East African population of 1 million persons (approximately 2/3 urban, 1.3 rural), with approximately 82,000 dogs (Table 1), use of vaccination Option 1 (50% of dogs are vaccinated against rabies each year), and assuming the high dog-dog rabies transmission scenario (see Table 2).

Reducing in the high transmission scenario the percent of dogs neutered, from 7.5% to 0%, during each vaccination program (50% dogs vaccinated, high rabies transmission scenario) causes the rise in dog rabies cases to start 1 year earlier (Fig 3). Neutering 20% of the dogs delays by 3 years, compared to the 7.5% dogs neutered, any increase in dog rabies cases (Fig 3).

**Fig 4:**
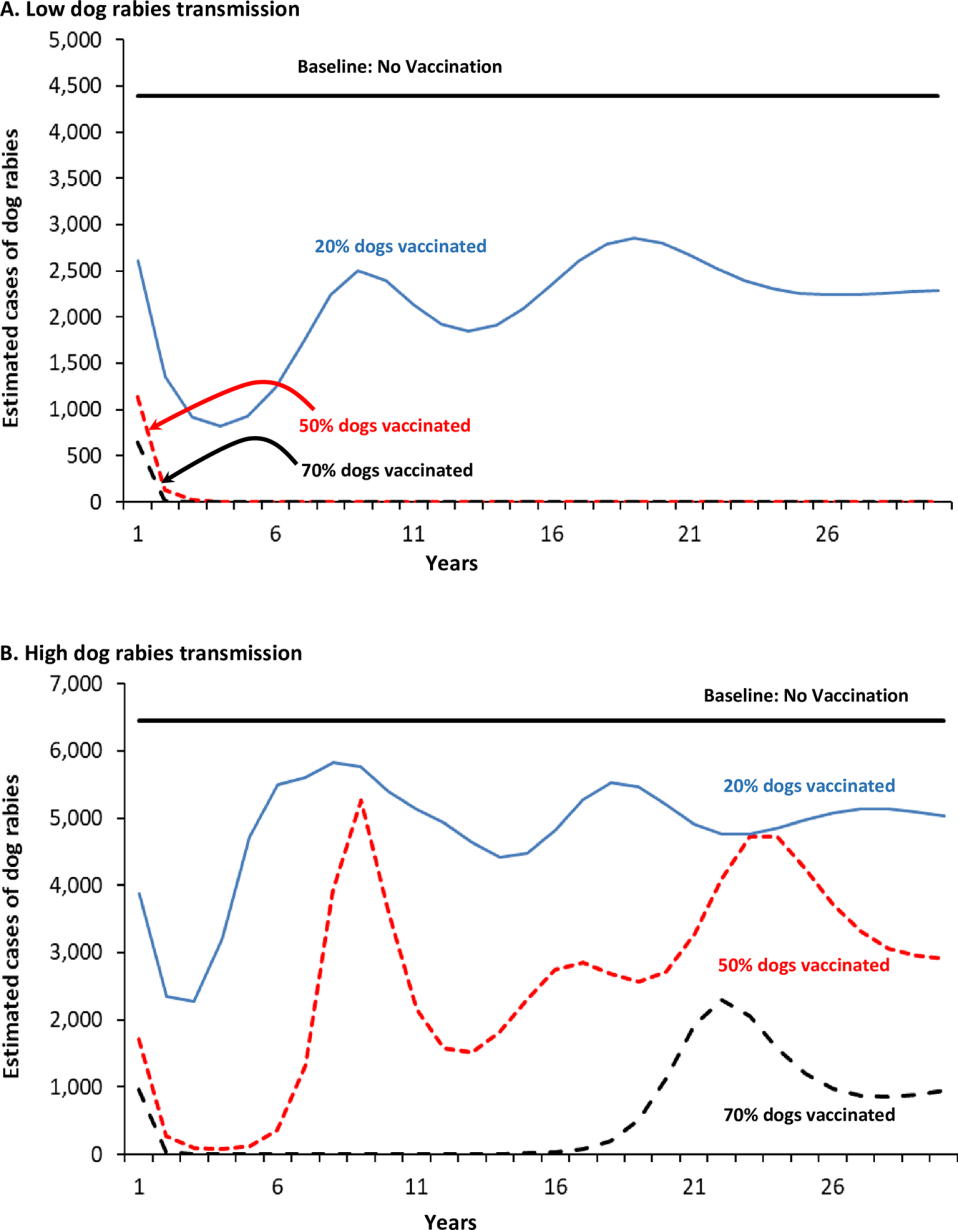
Sensitivity analysis: Cases of dog rabies for alternative annual dog rabies vaccination programs in East Africa: A. Low levels of transmission; B. High levels of transmission^a^ Footnotes: a. Results for dog rabies vaccination programs in an East African population of 1 million persons (approximately 2/3 urban, 1/3 rural), with approximately 82,000 dogs (Table 1). Rabies transmission risk is defined, in part, by the number of bites per rabid dog to another dog (see Table 2).

Comparing the impact of percentage of dogs vaccinated over 30 years, in a low dog rabies transmission scenario, both 50% and 70% vaccination rates essentially eliminate dog rabies within 3 years, and maintain that rabies-free state for 30 years (Fig 4). This assumes no re-introduction of rabies from outside the area in which dog vaccination programs are initiated. In contrast, with high dog-to-dog disease transmission, 50% dogs vaccinated will result in outbreaks of dog rabies at year 6, with cases occurring every year thereafter (Fig 4). An annual vaccination rate of 70% may result in an outbreak of rabies at approximately year 20. The importance of the level of dog rabies transmission (low versus high) is consistent with previous findings [8,12,30]. Further, due to the linear relationships in dog-to-human transmission built into RabiesEcon (S2 Appendix, Note #1), as the number of rabid dogs decreases, the number of human deaths will also proportionately decrease.

The impact of increasing PEP coverage from the base case of 21% to 99% is shown in Table 7. In the no vaccination scenario, increasing effective coverage to 99% greatly reduces the number of human deaths, over a 30 year span, from approximately 7,900 to approximately 100 (Table 7). Such coverage, however, increases costs to approximately $6.9 million. Because 50% dog vaccination, in the low transmission scenario, will effectively eliminate dog rabies with 10 years (Fig 1), it is feasible to assume that the dog vaccination program will, if not entirely cease, be greatly reduced. The no vaccination at 99% PEP coverage, while greatly reducing number of deaths, has to continue indefinitely because to risk of human rabies does not reduce. Thus, it may be more relevant to compare the $6.9 million costs of 30 year no vaccination, 99% PEP costs to the $1.8 million costs of 10 year dog vaccination, 99% PEP program (Table 7).

**Table 7:** Sensitivity analysis: Impact on number of human deaths due to dog rabies if assume 99% coverage of post-exposure prophylaxis (PEP); 10 and 30 year cumulative totals^a^.

	Cumulative totals			
	Baseline: No vaccination		Option 1: 50% dogs vaccinated	
	21% PEP	99% PEP	21% PEP	99% PEP
Rabid dogs				
Year 10	43,868	43,868	1,307	1,307
Year 30	131,605	131,605	1,307	1,307
Human deaths from canine rabies exposure				
Year 10	2,132	27	63	1
Year 30	7,887	101	63	1
Program costs (undiscounted)				
Year 10	392,241	1,766,440	1,368,394	1,784,819
Year 30	1,522,445	6,871,634	4,152,037	5,609,427
Cost per human death averted (undiscounted)				
Year 10	N/A^b^	N/A	451	Net Savings^c^
Year 30	N/A	N/A	336	Net Savings

a. Results produced using the low dog-to-dog transmission scenario (Table 2), 7.5% dogs neutered (Table 3)

b. N/A = not applicable because the “no vaccination” scenario is baseline.

c. Negative value of costs-per-human death avert signify net savings (government perspective).

**Fig 5:**
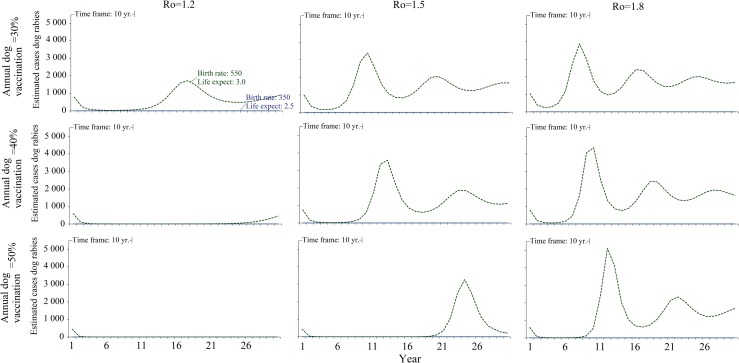
Sensitivity analyses: Impact in number of rabid dogs with changes in dog vaccination coverage, dog birth rate and life expectancy, and initial dog-to-dog rabies transmission. ^a^ Footnotes: a: Analyses run assuming 7.5% of dogs are neutered (Table 3), and using values for urban scenario (Table 1). Baseline values for the variables altered here are: Vaccination coverage 50%; Annual dog birth rate 676/1,000 dogs, dog life expectancy 3.0 years, and an Ro of 1.2.

When we simultaneously changed the 4 variables that most impact the number of rabid dogs, we found that the most important variables were the Ro, the dog birth rate, and dog -life expectancy (Fig 5). Whenever dog birthrate was cut from the baseline value of 676/ 1,000 dogs to 350/ 1,000 dogs, any level of vaccination included in the analyses eliminated dog rabies (Fig 5). However, combining higher levels of dog birth rate and life expectancy (550 births/ 1,000 dogs and 3.0 years) with higher levels of Ro (1.5 and 1.8) dog rabies may not be eliminated within 10 years (Fig 5). This suggests that dog rabies vaccination programs can benefit from any concurrent program that can effectively reduce dog birth rates. We note, however, that there are few examples from developing countries of such dog-population control programs being started and successfully maintained.

## Discussion

We estimate that vaccinating 20% (semi-annually) or 50% of an East African dog population will result in a cost-effectiveness of approximately $300–$450 per human death averted, and less than $10 per YLG. Our results were sensitive to the degree of dog-dog transmission (Fig 1 and Fig 5). For example, assuming that one infectious dog infects 1.2 other dogs allows our Option 2 (20% dogs vaccinated, semi-annually; low transmission scenario) to essentially eliminate dog rabies in a 10 year period. But, if it is assumed that one infectious dog infects 1.7 other dogs (+40% increase in risk of transmission; high transmission scenario), even vaccinating 50% of dogs annually is insufficient to eliminate dog rabies (though there would still be fewer rabid dogs than the no vaccination option). In the high transmissions scenario, it requires 70% of dogs vaccinated to eliminate dog rabies for at least 20 years.

Our results are similar to those of Bögel and Meslin, who found that dog vaccination, combined with administration of post-exposure prophylaxis to persons with a dog bite injury is more cost-effective than post-exposure prophylaxis alone [42]. Our estimates of the epidemiological impact of vaccinating 50% of the dog population are very similar to those of Coleman and Dye [12]. They used a mathematical model to estimate that dog rabies could be eliminated by vaccinating 39 to 57% of a dog population, with upper 95% confidence intervals of 55 and 71%, respectively [12]. They also estimated that achieving the WHO target of 70% of dogs vaccinated against rabies would give a 96.5% probability of preventing an outbreak. Zinsstag et al estimated that, in Chad, mass dog vaccination programs would result in a cost-effectiveness of $596 per human death averted in year 10 of a program (applying a 5% discount rate) [7]. Mindekem et al., reporting on dog rabies vaccination program in Chad run in 2012 and 2013, calculated a cost-effectiveness of $121 per Disability Life Year saved (when death is almost the only outcome from a case of human rabies, Disability Life Year saved and YLG are almost equivalent) [21]. It is noted that some have estimated higher Ro values than those we used (Table 2). Kitala et al estimated a higher value of 2.44 in Machokas District Kenya [16]. But, their 95% Confidence Interval of 1.52–3.36 spans the values that we used (Tables 1 and 6, Figs 1–5). In a separate paper, they stated that their higher incidences of dog rabies are “… probably both a function of better case reporting… and a very high relative incidence of disease” [43].

Our model and estimates have some limitations. There is the previously mentioned mechanics of the mathematical model that allows for the number of infectious dogs to be reduced to less than 1 (e.g., 0.5 infectious dog), but still able to transmit. However, users of RabiesEcon can easily ignore those “pop-up” outbreaks that occur in years well beyond the chosen analytic horizon (e.g., Figs 1 and 5). Another two important limitations are that the results can be, as demonstrated in the sensitivity analyses, greatly influenced by the values used to define the risk of dog-to-dog transmission (e.g., Fig 5). In many instances, public health official using RabiesEcon may not have ready access to reliable estimates from their locale for all the inputs required. The other important limitation is that, as a deterministic model, RabiesEcon does not contain any built-in uncertainty. Thus, to correct for such imitations, users of RabiesEcon are greatly encouraged to conduct extensive sensitivity analyses, with a primary aim to determine which variables most likely influence the outcomes of interest, and at what point changes in modeled outcomes may change public health decisions.

Other limitations derive from the fact that RabiesEcon calculates economic evaluations from the perspective of the government. Potential benefits accruing to others are not included. For example, Okell et al found that villagers in the Oromia region of Ethiopia considered rabies to be the zoonotic disease of greatest risk to both human and their livestock [44]. Jibat et al found that, in Ethiopia, rabies can cause a loss of 1–2 (range: 1–5) head of cattle in affected herds, and the value of such losses ranges from $147 up to $1,140, depending up the agricultural system (mixed crop-livestock or pastoral) [45]. In many parts of Africa, cattle are often sold at the end of their productive life. Their productive life includes being used for draft, which affects household income, labor and ultimately food security [46]. Thus, the value of cattle lost to rabies used by Jibat et al may be conservatively low. Public health decision makers, when using the results from RabiesEcon, will likely want to also consider including the value of such other benefits, even if they do not directly impact government budgets.

Programs designed to notably reduce, even eliminate, human dog rabies deaths have to rely on the expansion of dog rabies vaccination coverage. Human PEP does save lives, but it can be relatively expensive and it is difficult to ensure that all persons potentially exposed to dog rabies have timely access to PEP [6,34,41,47,48]. It may well be difficult to implement-and-maintain PEP programs over several years that achieve 99% coverage (Table 7). Expansion of dog rabies vaccination programs require local, political, and economic support [9, 49,50]. Anyiam et al have proposed a novel method to fund the required expansion of dog rabies vaccination programs [51]. They suggest that the government sell “development impact bonds” to private investors for the initial expansion. Assuming that the expanded vaccination program produces the anticipated results, then more traditional funding sources, such as the World Bank, African Development Bank, donor organizations, and the government can repay the bonds and continue funding the additional years of vaccination program. In this manner, banks, donors and the government only fund the program once a positive impact (i.e., success) has been demonstrated. It will require negotiations as to the premium needed by investors to accept the initial risk. To attract investors to such a funding scheme will require estimates of disease burden without intervention, costs of intervention, and impact of intervention. RabiesEcon can be used to provide such estimates.

Equally important to ensuring the success of the any dog rabies vaccination program is community involvement. The price of dog rabies vaccination to dog owners can notably reduce the willingness and/ or ability of dog owners to pay for dog vaccinations [31, 52]. Dog owners also have to understand the need to maintain the vaccination status of their dogs–reduction in cases of rabid dogs and human rabies deaths may lead to complacency, and thus increased risk of either an outbreak or a re-introduction of rabies (as modeled in Figs 1B, 4B and 5).

As dog rabies vaccination programs expand, and more dogs are vaccinated, there are other factors, beyond the current scope of RabiesEcon, which will need to be considered. These factors include the need for increased surveillance as cases of dog rabies decline. Such increased surveillance is needed to rapidly respond to any outbreak, or re-introduction, of dog rabies. It is possible that community involvement in such enhanced surveillance will be needed to ensure that such surveillance is successful [19, 40,43]. Further, as cases of dog rabies decrease, there will likely be a financial benefit to health care payers (e.g., government agencies) from improving the quality of screening human dog bite victims to receive PEP [34]. The goal of such screening would be to reduce the number of “false positives” (i.e., those who aren’t infected with rabies, but still receive PEP), whilst ensuring that there is no increase in the number of “false negatives” (i.e., those who are infected with rabies, but do not receive PEP).

In conclusion, as demonstrated by the example and results presented here, RabiesEcon can help translate the complex set of factors affecting dog rabies transmission and human deaths due to dog rabies into readily understood estimates of impact-of-vaccination and cost-effectiveness. RabiesEcon is sufficiently flexible that a user can enter the relevant data (Tables 1–5) from almost any country or locale, and thus estimate in costs-and-benefits of a dog rabies control program almost anywhere in the world. Such data may aid the expansion of dog rabies vaccination programs, and thus potentially aid the eventual elimination of dog rabies.

## Supporting information

S1 Appendix RabiesEcon: A tool to estimate the epidemiologic burden of dog rabies and potential epidemiologic and economic impact of a dog rabies vaccination program. This is a spreadsheet-based (Excel Microsoft Corp., Seattle, WA, 2016) tool.
(XLSX)

S2 Appendix Notes to accompany: Cost-effectiveness of dog rabies vaccination programs in East Africa. These are a set of additional Tables of input values and the mathematical equations used to build the RabiesEcon tool (S1 Appendix), and produce the results given in the main text.
(DOCX)
