# Supplementary material for: Cost-effectiveness of dog rabies vaccination programs in East Africa
Source: PLoS Negl Trop Dis. 2018 May 23;12(5):e0006490. doi: 10.1371/journal.pntd.0006490 (PMC5988334; doi:10.1371/journal.pntd.0006490)
Supplement: S2 Appendix — These are a set of additional Tables of input values and the mathematical equations used to build the RabiesEcon tool (S1 Appendix), and produce the results given in the main text. (DOCX) [file pntd.0006490.s002.docx]

**Supplementary material: Appendix S2**

Notes to accompany: Cost-effectiveness of dog rabies vaccination programs in East Africa

Rebekah H. Borse, Charisma Y Atkins, Manoj Gambhir, Eduardo A. Undurraga, Jesse D. Blanton, Jessie L. Dyer, Charles E. Rupprecht, Martin I. Meltzer^*^

*Corresponding Author:

Martin I. Meltzer, MS, Ph.D.

Mailstop C-18 Ph: 404-639-7778

1600 Clifton Rd.

Atlanta, GA 30333 e-mail: [MMeltzer@cdc.gov](mailto:MMeltzer@cdc.gov)

# Appendix S2.

**Supplemental Table 1:** Input values calculated by RabiesEcon to start model with initial run of 10,000 weeks to arrive at steady state^a^

| **Model Variable** | **Model inputs** | | **Source** |
| --- | --- | --- | --- |
|  | **Urban** | **Rural** |  |
| Effective reproduction number (R_0_) at start^b^ | 1.2150 | 1.0800 | Calculated^b^ |
| Susceptible dogs (dogs/ km^2^)^a^ | 166.0940 | 25.4919 | Calculated |
| Exposed dogs (dogs/km^2^)^a^ | 0 | 0 | Calculated |
| Infectious/rabid dogs (dogs/km^2^)^a^ | 0.0145 | 0.0003 | Calculated |
| Susceptible humans (humans/km^2^ ) | 3,007 | 189 | Calculated |
| Exposed humans (humans/km^2^) | 0 | 0 | Calculated |
| Infectious humans (humans/km^2^) | 0 | 0 | Calculated |
| Immune humans (humans/km^2^) | 0 | 0 | Calculated |
| Dog rabies infective period, life expectancy (days) | 5 | 5 | [[1](#_ENREF_1)] |
| Loss of vaccination immunity (wks 0-25) | .0081 | .0081 | [[2](#_ENREF_2)] |
| Loss of vaccination immunity (wks 26-52) | 0.1101 | 0.1101 | [[2](#_ENREF_2)] |
| Dog-human transmission rate (km^2^/dogs/week) | 0.00003 | 0.00040 | Calculated |
| Risk of clinical outcome per bite (rabid dog-dog) | 0.45 | 0.45 | [[1](#_ENREF_1)] |
| Dog rabies incubation period (days) | 45 | 45 | [[1](#_ENREF_1)] |

**Notes:**

a: The introduction of rabies into a previously rabies susceptible dog population initially causes great variation in the estimated weekly number of rabid dogs. RabiesEcon is programmed to take the user entered demographic and epidemiological information (Main text, Table 1), assume that a single rabid dog introduces rabies into a dog population, calculate the values shown in this Table, and then run an initial 10,000 weeks. This allows the weekly number of rabid dogs (before any interventions) to reach a steady state.

b: Infectious/Rabid dog proportion at start, T_0,_ is calculated first to determine the proportion of dogs entering the population that are infectious. The values used here are the equivalent of the “low transmission” scenario – Table 2, main text.

**Supplemental Table 2:** Input costs for the delivery of a mass dog rabies vaccination program (government perspective) targeting 50% of dogs in East Africa^a^

| **Item** | **Units** | **Work Days** | **Price/Unit (US dollars)** | **Total Cost (US dollars)** |
| --- | --- | --- | --- | --- |
| **1. Fixed costs^b^** |  |  |  |  |
| **Workers (per diem)** |  |  |  |  |
| Training Supervisor | 3 | 6 | 14.72 | 265 |
| Informational Supervisor | 0 | 0 | 0 | 0 |
| Vaccination Supervisor | 6 | 24 | 21.44 | 3,087 |
| Training Technician | 5 | 6 | 7.86 | 236 |
| Informational Technician | 15 | 24 | 11.92 | 4,291 |
| Vaccination Technician | 60 | 24 | 11.92 | 17,165 |
| Driver | 7 | 24 | 9.35 | 1,571 |
| Other Personnel |  |  |  |  |
| **Transportation** |  |  |  |  |
| Pick up (including gasoline) | 2 | 24 | 53.60 | 2,573 |
| Vehicle | 5 | 24 | 107.21 | 12,865 |
| Gasoline for Car | 80 | 24 | 1.07 | 2,058 |
| **Other vaccination program information** |  |  |  |  |
| Media (e.g. posters, leaflets, signs) | 7,500 | N/A | 1.56 | 11,700 |
| Additional equipment | 1 | N/A | 6,944 | 6,944 |
| **Total fixed costs** |  |  |  | **62,756** |
| **2. Variable costs** |  |  |  |  |
| **Percent vaccine wastage** |  |  |  | 10% |
| Vaccines | 41,113 |  | 0.65 | 26,723 |
| Syringes and needles | 41,113 |  | 0.11 | 4,522 |
| Vaccination certificates | 41,113 |  | 0.01 | 411 |
| Dog marking | 41,113 |  | 0.02 | 620 |
| **Total variable costs** |  |  |  | **35,504** |
| **3. Total** |  |  |  |  |
| Total dogs vaccinated |  |  |  | 41,113 |
| **Average cost per dog vaccinated** |  |  |  | **2.39** |

a: In the illustrative example, for a human population of approximately 1 million, there are 82,226 dogs (36,544 in urban setting, 45,682 in rural setting) (see Table 1, main text). We use this estimate (average cost per dog vaccinated when targeting 50% of dogs) as a baseline for various vaccination programs, assuming a linear relationship between fixed costs and the number of dogs vaccinated. Sources: Main text references;21,36,37,40.

b: These costs are “fixed” in the sense that they are the estimated, based on the referenced source, to be the resources needed to vaccinate

**Note #1: Equations used to define the dog rabies transmission model**

(source: Adapted from Zinsstag et al. [2])

For Glossary of Terms used in these equations, see Supplemental Table 3 (below)

$\frac{{dS}_{d}}{dt}=b_{d}N_{d}+\lambda_{d}R_{d}+(\sigma_{d}{(1-r}_{d})E_{d})-m_{d}S_{d}-\beta_{d}S_{d}I_{d}-{\gamma N}_{d}S_{d}-v_{d}\alpha_{d}S_{d}-c_{d}S_{d}$

$$\frac{{dE}_{d}}{dt}=\beta_{d}S_{d}I_{d}-m_{d}E_{d}-{\gamma N}_{d}E_{d}- (\sigma_{d}{(1-r}_{d})E_{d})-v_{d}\alpha_{d}E_{d}-\sigma_{d}r_{d}E_{d}-c_{d}E_{d}$$

$$\frac{{dI}_{d}}{dt}= \sigma_{d}r_{d}E_{d} -m_{d}I_{d}-{\gamma N}_{d}I_{d}-\mu_{d}I_{d}$$

$$\frac{{dR}_{d}}{dt}= (v_{d}{\alpha_{d}(S_{d}+E}_{d})) -m_{d}R_{d}-{\gamma N}_{d}R_{d}-\lambda_{d}R_{d}$$

$$\gamma=\frac{{(b}_{d}-m_{d})}{K}$$

**Equations used to define Dog to Human transmission model**

$$\frac{{dS}_{h}}{dt}={(b}_{h}(S_{h}+E_{h}+R_{h}))+\lambda_{h}R_{h}+\left( E_{h}\left( \frac{P2\left( 1-P6 \right)}{i_{head}}+\frac{P3\left( 1-P7 \right)}{i_{arm}}+\frac{P4\left( 1-P8 \right)}{i_{trunc}}+\frac{P5\left( 1-P9 \right)}{i_{leg}} \right) \right){-m}_{h}S_{h}-v_{h}\alpha_{h}S_{h}{-\beta}_{h}S_{h}I_{d}$$

$$\frac{{dE}_{h}}{dt}=\beta_{h}S_{h}I_{d}{-m}_{h}E_{h}-{P10\nu_{h}E}_{h}\left( \frac{P2P6}{i_{head}}+\frac{P3P7}{i_{arm}}+\frac{P4P8}{i_{trunc}}+\frac{P5P9}{i_{leg}} \right)-{(1-P10)E}_{h}\left( \frac{P2P6}{i_{head}}+\frac{P3P7}{i_{arm}}+\frac{P4P8}{i_{trunc}}+\frac{P5P9}{i_{leg}} \right) -E_{h}\left( \frac{P2\left( 1-P6 \right)}{i_{head}}+\frac{P3\left( 1-P7 \right)}{i_{arm}}+\frac{P4\left( 1-P8 \right)}{i_{trunc}}+\frac{P5\left( 1-P9 \right)}{i_{leg}} \right)$$

$$\frac{{dI}_{h}}{dt}=(1-P10)E_{h}\left( \frac{P2P6}{i_{head}}+\frac{P3P7}{i_{arm}}+\frac{P4P8}{i_{trunc}}+\frac{P5P9}{i_{leg}} \right){-m}_{h}I_{h}-\mu_{h}I_{h}$$

$$\frac{dR_{h}}{dt}=P10\nu_{h}E_{h}\left( \frac{P2P6}{i_{head}}+\frac{P3P7}{i_{arm}}+\frac{P4P8}{i_{trunc}}+\frac{P5P9}{i_{leg}} \right)+\nu_{h}\alpha_{h}S_{h}-m_{h}R_{h}-\lambda_{h}R_{h}$$

**Supplemental Table 3.** Glossary of terms used in RabiesEcon.

| Symbol | Variable | Estimate used |
| --- | --- | --- |
| T | *Time* | 1 week |
| *R_0_* | *Effective reproductive number at t_0_* | Calculated |
| *R_e_* |  | $\left( \frac{\sigma_{d}r_{d}\beta_{d}S_{d}}{\left( \sigma_{d}+m_{d} \right)\left( m_{d}+\mu_{d} \right)} \right)$ |
| S*_d_* | *Susceptible at t_0_* | Calculated |
| E*_d_* | *Exposed at t_0_* | 0% of N*_d_* dogs/ km^2^ |
| I*_d_* | *Infectious/Rabid at t_0_* | Calculated |
| R*_d_* | *Immune at t_0_* | 0% dogs/ km^2^ |
| N*_d_* | *Population at t_0_* | Set by user dogs/ km^2^ |
| *b_d_* | *Dog birth rate* | *Adjusted_birthrate/52/1000* week^-1^ |
| λ*_d1_* | Loss of vaccination immunity (weeks 0-25) | *Default as published in Zinsstag et al.[*[*2*](#_ENREF_2)*], 0.0081* week^-1^ |
| λ*_d2_* | Loss of vaccination immunity (weeks >26) | *Default as published in Zinsstag et al., 0.1101* week^-1^ |
| *Incubation* | *Dog rabies incubation period in days* | Set by user, default 45 days |
| *i_d_* | *Dog rabies incubation period in weeks* | Incubation/7.177 week |
| *σ_d_* | *Inverse of average incubation period* | 1/ *i_d_* |
| *r_d_* | *Risk of clinical outcome* | Set by user, default 0.45 |
| *Dog_life* | *Dog life expectancy* | Set by user, default 3 years |
| *m_d_* | *Death rate* | (1/dog_life)/52 week^-1^ |
| *β_d_* | *Transmission coefficient (inverse of time between dog contacts)* | $R_{0}$*($\left( \sigma_{d}+m_{d} \right)\left( m_{d}+\mu_{d} \right))/$ ($\sigma_{d}r_{d}S_{d}$) km^2^/(dogs ^. .^week) |
| γ | *Dog density dependent mortality* | *(b_d_ -m_d_ )/K* km^2^ / (dogs ^. .^week) |
| K | *Mean carrying capacity* | N*_d_*1.05*(1+1/(ln(*N*_d_*program area))* dogs km^2^ |
| *v_d_* | Dog vaccine efficacy | Set by user, default 0.95 |
| *vaccine* | Vaccination coverage per program | Set by user |
| *α_d_* | Dog vaccination rate (weeks 1-10) | -(1/10)*(*ln*(1-vaccine) week^-1^ |
|  | Dog vaccination rate *(weeks 11+)* | 0% week^-1^ |
| *c_d_* | Culling rate per program | 0% |
| Infective | *Dog rabies infective period* | Set by user |
| μ*_d_* | *Inverse of average infective period, rabid mortality rate* | (1/infective)*7 week^-1^ |
| S_h_ | *Susceptible at t_0_* | N_h_ humans/ km^2^ |
| E_h_ | *Exposed at t_0_* | 0 humans/ km^2^ |
| I_h_ | *Infectious/Rabid at t_0_* | 0 humans/ km^2^ |
| R_h_ | *Immune at t_0_* | 0 humans/ km^2^ |
| N_h_ | *Population at t_0_* | Set by user humans/ km^2^ |
| *birth_rate* | *Human birth rate per 1,000 population* | Set by user |
| *b_h_* | *Human birth rate* | (birth_rate/52)/1000 week^-1^ |
| λ*_h_* | Human loss of vaccination immunity rate | 0 week^-1^ |
| *Life_expectancy* | *Human life expectancy* | Set by user |
| *m_h_* | *Human mortality rate* | (1/life_expectancy)/52 week^-1^ |
| *v_h_* | Human vaccine efficacy | Set by user, default 0.95 |
| *α_h_* | Human pre exposure prophylactic rate | 0 week^-1^ |
| *Human_deaths* | *Human deaths annually in current program* | Set by user |
| *β_dh_* | *Dog human transmission rate, based on value in Zinsstag et al 2009* | Set by user, default 0.0002054 km^2^/(dogs ^. .^week) |
| *P10* | Human post exposure prophylactic (PEP) vaccination rate | Set by user week^-1^ |
| *P2* | Probability of a bite to the head | Set by user, default 0.07 [[3-5](#_ENREF_8)] |
| *P3* | Probability of a bite to the arm | Set by user, default 0.384 [3-5] |
| *P4* | Probability of a bite to the trunk | Set by user, default 0.06 [3-5] |
| *P5* | Probability of a bite to the leg | Set by user, default 0.486 [3-5] |
| *P6* | Prob. of developing rabies after a bite to the head | Set by user, default 0.45 [3-5] |
| *P7* | Prob. of developing rabies after a bite to the arm | Set by user, default 0.275 [3-5] |
| *P8* | Prob.of developing rabies after a bite to the trunk | Set by user, default 0.05 [3-5] |
| *P9* | Prob.of developing rabies after a bite to the leg | Set by user, default 0.05 [3-5] |
| *i_head_* | Human incubation period after bite to the head | Set by user, default 15.00 days |
| *i_arm_* | Human incubation period after bite to the arm | Set by user, default 15.00 days |
| *i_trunk_* | Human incubation period after bite to the trunk | Set by user, default 20.00 days |
| *i_leg_* | Human incubation period after bite to the leg | Set by user, default 45.00 days [[1](#_ENREF_1)] |
| Infective_h | *Average infective period for humans* | 7 days |
| μ*_h_* | *Inverse of average infective period, rabid human mortality rate* | (1/infective_h)*7 week^-1^ |
| *pups_d_* | Number of pups per litter | Default, 4 |
| *Dog_births* | Dog birth rate (per 1,000 dogs, per year) | Set by user |
| *births_d_* | Number of dogs born annually at t_0_ | (Dog_births/1000)*N*_d_* |
| *litters_d_* | Average annual number of litters at t_0_ | *births_d_* /pups*_d_* |
| *Adult_ female_d_* | Number of adult female dogs t_0_ | N*_d_* /2 |
| *whelped_d_* | Number of female dogs whelped in last 6 months | *(litters_d_ / Adult_ female_d_)*(1- spay_d_)*(1- neuter_d_)* |
| *spay_d_* | Proportion of adult female dogs spayed | Set by user |
| *neuter_d_* | Proportion of adult male dogs neutered | Set by user |
| *Adjusted birthrate_d_* | Adjusted dog birth rate (per 1000 dogs) | 1000*( *whelped_d_* * *Adult_ female_d_* * *pups_d_* /N*_d_*) |

Source: Zinsstag et al [2].

**Note #2: Calculation of Years of Life Gained (YLG) and cost-effectiveness of YLG**

$$Cost per Year of Life Gained (YLGs)= \frac{Costs of dog vaccination program-costs incurred with no vaccination program}{Number of Years of Life Lost without vacciantion program-Years of Life Gained with vaccination program}$$

**Where:** We calculated YLGs, in RabiesEcon, using the formula for Years of Life Lost, as presented by Mathers et al [11].

**
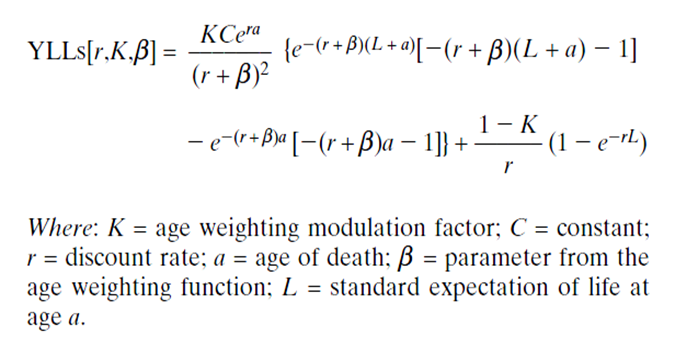
**Where, in RabiesEcon, we used the input values in Supplemental Table 4 to calculate YLL/ YLG.

We did not include any disability, or loss of life years, associated with treatments for dog bites and post-exposure prophylaxis (PEP). Patients definitely do experience disabilities due to receiving such treatments. However, given that a loss of life that is essentially inevitable if a patient develops a clinical case of rabies, it is the YLG from preventing such losses that are the prime focus of public health officials.

| **Supplemental Table 4:** Parameters for Years of Life Lost/ Gained | |
| --- | --- |
| a, Average age of death (human) due to rabies exposure (years) | 10.00 |
| Life expectancy at 10 years | 58.5 |
| L, standard expectation of life at age a | 48.50 |
| K, age weighting modulation factor | 1 |
| β, parameter from the age weighting function | 0.02 |
| C, Constant | 0.0634 |
| **Years of Life Lost (YLL) per death, at 3% discounting** | **23.9** |
|  |  |
| **Years of Life Lost (YLL) per death, at 0% discounting** | **49.0** |
| **Years of Life Lost (YLL) per death, at 16% discounting** | **4.5** |

# References for the Appendix S2

1. Hampson K, Dushoff J, Cleaveland S, Haydon DT, Kaare M, et al. (2009) Transmission Dynamics and Prospects for the Elimination of Canine Rabies. Plos Biol 7: 462–471.

2. Zinsstag J, Dürr S, Penny M, Mindekem R, Roth F, Gonzalez SM, et al. Transmission dynamics and economics of rabies control in dogs and humans in an African city. Proc Natl Acad Sci. 2009; 106(35):14996-5001.

3. Babes V. Treatise on rabies (translated from French). Paris: Baillière et Fils. (Cited in Baer et al. 1990); 1912.

4. Baer G, Bellini W, Fishbein D. Rhabdoviruses. In: Fields B, editor. Virology. 2nd ed. New York: Raven Press; 1990.

5. Cleaveland S, Fevre EM, Kaare M, Coleman PG. Estimating human rabies mortality in the United Republic of Tanzania from dog bite injuries. Bull WHO. 2002; 80(4):304-10.

6. Mathers CD, Salomon JA, Ezzati M, Begg S, Van der Hoorn S, and Lopez AD. Sensitivity and Uncertainty Analyses for Burden of Disease and Risk Factor Estimates. Global Burden of Disease and Risk Factors, ed., 399-426. 2006. New York: Oxford University Press
